# Development and evaluation of a survey instrument to assess veterinary medical record suitability for multi-center research studies

**DOI:** 10.3389/fvets.2022.941036

**Published:** 2022-08-05

**Authors:** Dora Praczko, Amanda K. Tinkle, Crystal R. Arkenberg, Robyn L. McClelland, Kate E. Creevy, M. Katherine Tolbert, Brian G. Barnett, Lucy Chou, Jeremy Evans, Kellyn E. McNulty, Dog Aging Project Consortium, Jonathan M. Levine

**Affiliations:** ^1^Department of Small Animal Clinical Sciences, College of Veterinary Medicine and Biomedical Sciences, Texas A&M University, College Station, TX, United States; ^2^Department of Biostatistics, University of Washington, Seattle, WA, United States

**Keywords:** electronic medical record, inter-rater agreement, clinical trial, verification, point score

## Abstract

Here we describe the development and evaluation of a survey instrument to assess the research suitability of veterinary electronic medical records (EMRs) through the conduct of two studies as part of the Dog Aging Project (DAP). In study 1, four reviewers used the instrument to score a total of 218 records in an overlapping matrix of pairs to assess inter-rater agreement with respect to appropriate format (qualification), identification match (verification), and record quality. Based upon the moderate inter-rater agreement with respect to verification and the relatively large number of records that were incorrectly rejected the instrument was modified and more specific instructions were provided. In study 2, a modified instrument was again completed by four reviewers to score 100 different EMRs. The survey scores were compared to a gold standard of board-certified specialist review to determine receiver operating curve statistics. The refined survey had substantial inter-rater agreement across most qualification and verification questions. The cut-off value identified had a sensitivity of 95 and 96% (by reviewer 1 and reviewer 2, respectively) and a specificity of 82% and 91% (by reviewer 1 and reviewer 2, respectively) to predict gold standard acceptance or rejection of the record. Using just qualification and verification questions within the instrument (as opposed to full scoring) minimally impacted sensitivity and specificity and resulted in substantial time savings in the review process.

## Introduction

Electronic medical records (EMRs) have been critical to the expansion of veterinary clinical research, and their widespread use has coincided with growth in multi-institutional studies, large retrospective case series, and biobank-driven research. Studies have been conducted using the Veterinary Medical Database started by the National Cancer Institute which provides a summary of electronic records from a select group of North American Veterinary Schools (1), along with electronic record databases housed in the United Kingdom (Small Animal Veterinary Surveillance Network), and the Royal Veterinary College (VetCompass) (2, 3). Having access to these records allows researchers to form conclusions based on larger, more diverse groups of animals. Multi-center databases created from EMRs allow for more variability in location, disease prevalence, financial status and the type of practice visited (1). Furthermore, data from EMRs is amenable to extraction by machine learning and natural language processing techniques which facilitates large-scale research (4, 5).

Despite the wide use of EMRs in veterinary research, there are limited data on how to appropriately sort, manage, and screen these EMRs to determine whether the information provided represents a medical record suitable for the clinical study at hand (6). To be suitable, an EMR must be confirmed to represent the correct patient and must contain a minimum set of clinical data relevant to the objectives of the research. In the Dog Aging Project (DAP), a multi- institutional citizen scientist collaborative, participating owners obtain and submit their dog's EMRs. The DAP is an initiative that aims to understand how genes, lifestyle, and environment influence aging. The goal is to use this information to increase animals' period of life spent free from disease. Currently, there are over 33,000 dogs enrolled in the project with a goal of enrolling 100,000 dogs. Owners are able to nominate their dog to participate in the DAP by completing a comprehensive survey on the website and optionally submitting their EMRs. Information from the EMRs is used to sort some participants into smaller nested cohorts in which participation of a veterinarian may be required to collect samples.

Here we describe the iterative development and validation of a survey instrument to assess the suitability of these participant-submitted EMRs. The survey was assessed with respect to inter-rater agreement amongst four individuals (veterinarians and paraprofessionals) across a range of questions that determined validity of data entered and quality of information. An optimal EMR survey-based cut-score was developed by comparing survey data to board-certified specialist review, which was considered the gold standard.

## Materials and methods

The Dog Aging Project (DAP) has been described (7). Briefly, the DAP interacts with participants primarily through personalized online portals where research tasks are presented. All owners complete an initial comprehensive questionnaire about their dog's husbandry, environment and health, called the Health and Life Experience Survey (HLES), which is updated annually. In the HLES, each participant is asked to identify their primary care veterinarian, and to indicate whether or not they are willing to share their dog's medical records with the DAP for research purposes. Participants who indicate willingness to share their dogs' medical records are invited to obtain EMRs from their primary veterinarian and submit them using one or more of the following formats:.pdf,.txt,.doc,.docx, or.rtf. The study data from the DAP are collected and managed using REDCap (Research Electronic Data Capture) tools hosted at the University of Washington (8, 9).

Two studies were conducted sequentially to assess and refine a survey instrument that would allow personnel to determine that the submitted information was an EMR, was for the correct participant, demonstrated the existence of a Veterinary-Client-Patient Relationship (VCPR) between the primary care veterinarian and the participant ensuring the patient had been seen by a veterinarian within the last 12 months and contained a basic set of information about the participant (10). The gold standard to which surveys were compared was independent, subjective record review by two board-certified internists (KEC, MKT). The board-certified internists rated a record as “pass” or “fail” after review of available contents. The board-certified internists were instructed to pass records that would be considered an appropriate, informative veterinary record for the correct participant if they were seeing the patient in a referral clinical setting. The reason for failing a record was recorded (incorrect record, unclear if correct record, insufficient clinical information, insufficient number of entries for dog's age or insufficient VCPR, invoice information only, and other) for purposes of facilitating discussion of any disagreement.

### Study 1

A survey instrument was developed by a multidisciplinary team within the DAP to assess whether a submitted EMR established the presence of an active VCPR and was sufficiently rich in data to represent a valid medical record (Supplementary Table 1). Information gathered from the EMRs served two purposes: determine the quality of the medical record such that it could be confirmed a true medical record and gather basic information about the dog to be used for cohort enrollment and other research purposes. Qualification and verification were used to ensure the record could be easily processed and that the patient/client identity matched HLES data. Qualification and verification criteria were chosen to correspond with basic information legally required to be included in a medical record according to the Texas Administrative Code §573.52. Once a record was determined to be appropriate for review, quality points were assigned based upon the presence of key information, including date of birth, sex (including spay/castration status), body weight, records of medical procedures, recency of data entry, preventative health care visits, and other factors. These quality questions aimed to determine the amount of detail present in the records, with a goal of determining the strength of data obtained from the EMRs. Four individuals (veterinarians and veterinary technicians) reviewed EMRs using the survey. The survey instrument was assessed based upon inter-rater agreement and mean (standard deviation) time required for reviewers to complete the survey. Parallel to this, board-certified individuals reviewed records to generate data including pass/fail status of records and rater agreement as to pass/fail status.

The first 218 records submitted by participants were included for review in Study 1. The four individuals completing survey of records were grouped into a matrix of 6 unique pairs (see Supplementary Table 2), with one individual in the pairing being assigned as “Reviewer 1” and the second as “Reviewer 2.” Each individual reviewer assessed 109 records in total, broken up into sets of 36 or 37 records with each pairing. This paired, matrix arrangement facilitated agreement analysis and allowed for the entire cohort of 218 records to be surveyed twice. Reviewers were instructed to stop scoring a record and rate it “unacceptable” (0 points) if the record was not at least 90% digital and/or not legible to read (qualification questions). A threshold of >90% digital was chosen to avoid excluding otherwise eligible EMRs because they additionally contained hand- written items such as vaccine certificates or anesthesia monitoring sheets. Records that met the qualification criterion were then reviewed in a second step using verification questions to ensure that the record matched the HLES data exactly (dog name, owner last name, owner home ZIP code), with reviewers instructed that inexact matches should result in an “unacceptable” rating (0 points). Reviewers recorded the time elapsed to complete the survey instrument. The two board-certified internists determined pass/fail status for the same cohort of 218 EMRs receiving quality point scores by survey, with one specialist assessing 139 and the other specialist assessing 145. The overlap in records assessed allowed for inter-rater agreement to be determined between specialists.

### Study 2

Study 2 was conducted following analysis of data from study 1 and a meeting among all raters to identify points of confusion or discrepancy and agree on measures that would make the instrument more clear. The quality scoring instrument was modified to increase the specificity of questions, and standardized training was provided to reviewers to eliminate inconsistencies in processing that were noted in study 1. The specificity of questions was increased by further clarifying and breaking down questions after assessing discrepancies between reviewers in study 1. Reviewers also received an interactive virtual training where the questions used to score the EMRs were discussed in detail and a written guide that addressed any known situations or variations that could cause confusion. The goals of study 2 were to rigorously evaluate the modified scoring instrument after standardized training and to evaluate whether numeric quality scoring was necessary to achieve agreement with board-certified internist assessment of EMR pass/fail. The study 1 survey questions were modified slightly but consisted of the same three groups: verification, qualification, and EMR quality (Supplementary Table 3). Qualification questions ensured the EMR met minimum standards for usability by DAP staff and included assessment regarding digital format (>90% typed), legibility, completion in the English language, and at least one veterinary visit within the past 2 years. Verification questions concerned patient and client identifiers in the EMR and explored whether these *reasonably* matched identifiers clients had submitted to the DAP in HLES; minor variations in spelling of dog name or owner last name were tolerated (e.g., differences of 1–2 letters that did not change the overall meaning of the name), and either owner ZIP code or phone number could be used as verification of contact information. Other questions from study 1 concerning the quality of the EMR were included following the qualification and verification tiers. Written instructions were embedded within the survey instrument, to define appropriate responses to questions and potentially improve rater agreement. The process for pass/fail review by board-certified internists was identical to study 1.

A new set of 100 records were scored by four reviewers, two of whom had participated in study 1. Each record was scored by two reviewers, and all possible pairs were represented in a matrix similar to study 1 so that inter-rater agreement could be analyzed as in study 1. Raters were instructed *via* embedded instructions and in print to stop scoring a record and rate it “unacceptable” (0 points) if they could not answer affirmatively to all verification and qualification questions. Reviewers were instructed to record the time required to complete the verification and qualification tiers and the entire survey instrument. As in study 1, two board-certified internists evaluated the same 100 records, each scoring 60 records pass/fail. The overlap of 20 records allowed for inter-rater agreement between the specialists to be calculated.

### Statistical analysis

There were five components determining whether a record would move on to full numeric quality scoring: whether the record was digital, whether the submitted record was one of the correct file types, whether the record was legible, whether the record corresponded to the correct dog, and whether the dog was seen in the clinic in the prior 2 years. These five binary components, along with a composite indicating whether the record passed all criteria, were evaluated individually for chance-corrected agreement between the two reviewers using Kappa statistics. Kappa values were interpreted as follows: <0 = no agreement, 0.0–0.2 = slight agreement, 0.21–0.4 = fair agreement, 0.41–0.6 = moderate agreement, 0.61–0.8 = substantial agreement, 0.81–1.0 = almost perfect agreement (11). The quality scores (range 0–15) were visually compared between reviewers *via* a scatterplot, summarized as mean absolute difference, and evaluated *via* the intra-class correlation coefficient. Area under the ROC curve was used to summarize how well the reviewers were able to discriminate between records classified as acceptable by the board-certified internist expert reviewer pass/fail score, which was considered the gold standard. The optimal cut point to separate an acceptable record from an unacceptable record (as assessed by the pass/fail gold standard) was determined by maximizing Youden's Index (the sum of sensitivity and specificity).

## Results

### Study 1

Board-certified reviewers passed 72% (157/218) of EMRs. Medical records received failing ratings due to insufficient clinical information (23/61), insufficient VCPR (11/61), invoice information only (13/61), no digital records (25/61), and other (23/61). Twenty EMRs were reviewed by both board-certified reviewers and there was agreement as to pass/fail rating in 95% (19/20) of instances (kappa 0.83).

A total of 29/218 (13.1%) failed the 90% digital format requirement as assessed by both reviewers, and 18/218 (8.3%) records had unacceptable format according to 1 reviewer. The interrater reliability for formatting eligibility (qualification) was substantial (Kappa 0.71, SE 0.068). A total of 171/218 (78.4%) records were then eligible for patient/client identifier verification. A total of 20/171 (11.7%) failed identifier verification by both reviewers, and for 27/171 (15.8%) records, one reviewer believed the record failed ID verification. The interrater reliability was moderate for identifier verification (kappa 0.5, SE 0.077). There were 123/218 (56.4%) records that passed both qualification and verification steps.

The 123 records that passed both qualification and verification steps by 2 reviewers were assessed by pairs of non-board-certified reviewers, using the survey instrument, to generate a quality point score. The average time to process records was 9 min (S.D. 4.4 min). Of the 123 records assigned quality scores by two reviewers, reviewer scores matched in 30/123 (24.6%) instances, 78/123 (63.9%) records had a one-point difference in reviewer quality score, 10/123 (8.2%) had a two-point difference, and 5/123 (4.1%) records had a > two-point difference. The median absolute difference was one point (range, 0–13). The intraclass correlation coefficient amongst all reviewers was 0.77 (95% CI 0.69 to 0.84).

### Study 2

In this study, the agreement was reviewed for each qualification and verification question (Table 1). The question with the lowest kappa of 0.53 addressed whether a veterinarian had seen the dog within the last 2 years. For this question, reviewers disagreed on 10/100 (10%) records. Reviewers fully agreed on the file type and achieved a Kappa of 1. For the remaining questions, regarding the legibility of the record, whether the primary clinic provided digital records and whether all record criteria were verified, Kappa scores ranged between 0.76 and 0.88. Records that received a point score of zero had a mean processing time of 2.4 min (S.D. 2.6 min), while those that had point scores > 0 had a mean processing time of 11.5 (SD of 5.7).

**Table 1:** Inter-rater agreement amongst four individuals for each qualification question and record verification in study 2.

	**# Evaluated by Both Processors**	**Processor agreement**			**Percent agreement**	**Kappa**
		**Agree Yes *N***	**Agree No *N***	**Disagree *N***		
Primary clinic record digital (Qualification)	100	83	13	4	96%	0.84
Correct file format (Qualification)	100	92	8	0	100%	1
Record legible (Qualification)	100	84	13	3	97%	0.88
Record met all verification criteria	100	79	14	7	93%	0.76
Dog seen at clinic within past 2 years (Qualification)	100	83	7	10	90%	0.53
Include record?	100	75	20	5	95%	0.86

**Figure 1:**
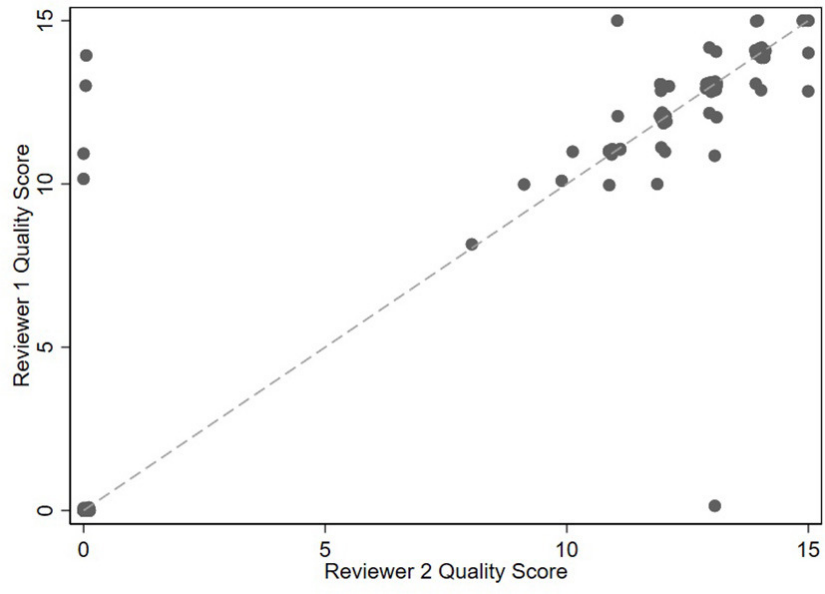
Scatterplot of 100 medical record scores between generated by 4 individuals grouped into reviewer 1 and reviewer 2 categories in study 2. Unverified records are assigned a zero score.

There were 76/100 (76%) medical records in which both reviewers agreed that the record met qualification and verification standards. Comparing the two reviewers who looked at each record, there was a zero-point difference for 52/76 (68.4%) records, a one-point difference for 20/76 (26.3%) records, a two-point difference for 4/76 (5.3%) records, and a > two-point difference for 0/76 (0%) records (Figure 1). The median point difference was zero points (range, 0–14), and the ICC was 0.84 (95% CI 0.76 to 0.90).

A total of 78/100 records were passed by gold standard, board-certified reviewers. The board-certified reviewers both evaluated 20 records and agreed on pass/fail status on 19 of these (kappa 0.83, CI 0.5–1). For the first round of review, the ROC area under the curve was 0.93 (95% CI 0.88 to 0.99). The optimal cut-off based on Youden's index was determined to be between 8 and 9 points, where the sensitivity would be 0.96 and the specificity 0.82. Using a cutoff of >8, 79/100 records would be accepted, with four records being accepted that were failed by gold standard review and three records being rejected that were passed by gold standard review. If zero was used at the cut-off, the sensitivity would remain 0.96, and the specificity would be 0.77. The ROC area under the curve for the second round of reviews was 0.95 (95% CI 0.9 to 0.99). The optimal cut-off based on Youden's index was determined to be 8.5 points, where the sensitivity would be 0.95, and specificity would be 0.91. If a cutoff of > 8 were used, 76 records would be acceptable with two records being accepted that were failed by gold standard review and four records being rejected that were passed by gold standard review. If zero were to be used as the cut-off, sensitivity would remain 0.95, and specificity would be 0.86.

## Discussion

Research groups that utilize complex clinical data should ensure that EMRs are suitable for inclusion in studies. This becomes especially important in complex, multi-institutional trials where EMRs come from multiple sources in divergent formats. We developed a survey instrument to assess the validity of patient data and the quality of the EMR. While the final version of the instrument had strong inter-rater agreement and correlated well with our gold standard, it was time consuming to complete and required extensive training. Therefore, the DAP currently uses only qualification and verification questions which are easy to deploy, require minimum time, and had nearly identical ROC characteristics compared to the full survey instrument.

Participants whose EMRs do not pass qualification are included in the DAP but are not assigned to any cohorts that involve the collection of samples from their dogs. The owners are not notified of their EMR “failing” and continue to receive invitations to participate in all online components of the DAP. Additionally, owners are invited to upload an updated EMR annually, which may allow some owners who could not accomplish the task in the initial year to complete it successfully in later years. The most common reasons for a record failing to qualify include the participants uploading invoices instead of EMRs or the records not being digital. Discrepancies in last names or contact information were less common reasons for failure, perhaps because participants can indicate multiple owners on their DAP profiles and can update their phone numbers, addresses, or zip codes in their DAP profile at any time.

In the future, the DAP plans to develop automated processes for the extraction of data from EMRs, as other investigators have done, which greatly increases the efficiency of large- scale research (4, 5). As the DAP EMRs in the study presented here are submitted by participants rather than directly harvested from veterinary clinics, establishing that submitted EMRs are correctly associated with the study participants and contain a minimum set of data is necessary before EMRs can be associated for use in the development of extraction algorithms.

In study 1, a large number (43.6%) of EMRs did not meet survey qualification verification steps and there was only moderate inter-rater agreement with respect to EMR verification (e.g., Kappa 0.5 for identifiers). Board-certified experts rejected fewer records (28%) and had much more substantial agreement as to record “pass” or “fail” status. We believe that the limited printed guidance associated with the survey and the lack of formal reviewer training prior to survey use may have affected inter-rater agreement and contributed to the larger number of records that did not pass qualification and verification. In general, structured rater instructions and training improve agreement and validity in a variety of assessments, ranging from radiographic or other clinical measurements to those relying on survey instruments (12, 13). When looking at the 123 records that did pass qualification and verification questions by both reviewers, there was substantial inter-rater agreement with respect to total survey quality points.

Based upon findings in study 1, we provided enhanced instructions and rater training for those individuals completing surveys in study 2. The survey instrument was likewise restructured to improve workflow and questions were re-worded to enrich the information that could be collected. Providing further training showed clear improvement, increasing the inter-rater agreement from 0.77 to 0.84 (13). Inter-rater agreement was substantial across most qualification and verification questions, including those that concerned patient identification. Those completing the surveys rejected 24% of records during the qualification and verification steps. The percentage of records rejected in study 2 was almost identical between the non-board-certified reviewers (24%) and the board-certified experts (22%). It is unclear if additional training could have increased inter-rater agreement further. Board-certified specialists agreed on all but one EMR. This record was failed by one of the specialists due to missing information from the veterinary clinic indicated as the primary clinic; the submitted EMR included records from specialist referrals. Both board-certified specialists agreed the EMRs provided were in fact medical records, however the EMR was failed after discussion.

In this study, we compared total quality point scores from two rounds of review to gold standard board-certified pass/fail status to determine an optimal cut-off that would replicate the board-certified review assessment. An important objective of the study was to identify a review instrument that could be used by trained staff members rather than requiring veterinarians to review all submitted EMRs, as the review of the thousands of EMRs obtained by the DAP will be time consuming. We were able to determine cut-off values with sensitivities of 96% (first round) and 95% (second round) and specificities of 82% (first round) and 91% (second round). Using a cut-off of >0 minimally impacted sensitivity and specificity and would obviate the need to complete steps beyond simple record qualification and verification questions. In addition, the point system was an extremely time-consuming method for scoring the records (14, 15). The mean time required to complete qualification and verification questions only (based upon those records that received a score of 0 and did not move beyond this step) was 2.4 min (SD 2.6), whereas the mean time to complete the entire survey was 11.5 min (SD 5.7). Currently, the DAP deploys only qualification and verification questions to screen records due to the minimal disadvantage with respect to sensitivity and specificity and the substantial time savings per record.

## Conclusion

We developed a survey instrument that allows trained individuals to assess the usability of EMRs for research and compared the survey to gold standard review by board-certified veterinarians. We established cut-off scores for qualification and verification questions that had a high sensitivity and specificity for predicting acceptance of EMRs by gold standard review. Other multi-center studies can screen their EMRs with methods developed in this paper. Researchers can choose to utilize the full instrument to identify usable records or the shortened system to quickly identify usable EMRs. Currently, the DAP uses methods described here to screen thousands of EMRs for usability in studies.

## Data availability statement

The original contributions presented in the study are included in the article/Supplementary material, further inquiries can be directed to the corresponding author.

## Author contributions

DP, AT, and JL wrote the initial draft of this paper. CA, KC, MT, AT, BB, LC, JE, and KM developed, pilot-tested, and revised the eligibility instrument. RM performed analysis of the study data. All authors, including Consortium authors, have been involved in the design and implementation of the DP, and they have had the opportunity to participate in editing both form and content of this paper and have approved the final version.

## Conflict of interest

The authors declare that the research was conducted in the absence of any commercial or financial relationships that could be construed as a potential conflict of interest.

## Publisher's note

All claims expressed in this article are solely those of the authors and do not necessarily represent those of their affiliated organizations, or those of the publisher, the editors and the reviewers. Any product that may be evaluated in this article, or claim that may be made by its manufacturer, is not guaranteed or endorsed by the publisher.

## References

[1] Bartlett PC Van Buren JW Neterer M Zhou C. Disease surveillance and referral bias in the veterinary medical database. Prev Vet Med. (2010) 94:264–71. 10.1016/j.prevetmed.2010.01.007 20129684

[2] Paynter AN Dunbar MD Creevy KE Ruple A. Veterinary big data: when data goes to the dogs. Animals. (2021) 11:1872. 10.3390/ani11071872 34201681 PMC8300140

[3] McGreevy P Thomson P Dhand NK Raubenheimer D Masters S Mansfield CS. VetCompass Australia: a national big data collection system for veterinary science. Animals. (2017) 7:74. 10.3390/ani7100074 28954419 PMC5664033

[4] Hur B Hardefeldt LY Verspoor K Baldwin T Gilkerson JR. Using natural language processing and VetCompass to understand antimicrobial usage patterns in Australia. Aust Vet J. (2019) 97:298–300. 10.1111/avj.12836 31209869

[5] Jones-Diette JS Dean RS Cobb M Brennan ML. Validation of text-mining and content analysis techniques using data collected from veterinary practice management software systems in the UK. Prev Vet Med. (2019) 167:61–7. 10.1016/j.prevetmed.2019.02.015 31027723

[6] Worster A. Advanced statistics: understanding medical record review (MRR) studies. Acad Emerg Med. (2004) 11:187–92. 10.1111/j.1553-2712.2004.tb01433.x 14759964

[7] Creevy KE Akey JM Kaeberlein M Promislow DEL Dog Aging Project C. An open science study of ageing in companion dogs. Nature. (2022) 602:51–7. 10.1038/s41586-021-04282-9 35110758 PMC8940555

[8] Harris PA Taylor R Thielke R Payne J Gonzalez N Conde JG. Research electronic data capture (REDCap)–a metadata-driven methodology and workflow process for providing translational research informatics support. J Biomed Inform. (2009) 42:377–81. 10.1016/j.jbi.2008.08.010 18929686 PMC2700030

[9] Harris PA Taylor R Minor BL Elliott V Fernandez M O'Neal L. The REDCap consortium: Building an international community of software platform partners. J Biomed Inform. (2019) 95:103208. 10.1016/j.jbi.2019.103208 31078660 PMC7254481

[10] The veterinarian-client-patient relationship (VCPR): American Veterinary Medical Association. (2012). Available online at: https://www.avma.org/resources-tools/pet-owners/petcare/veterinarian-client-patient-relationship-vcpr

[11] Landis JR Koch GG. The measurement of observer agreement for categorical data. Biometrics. (1977) 33:159–74. 10.2307/2529310 843571

[12] Bergstrom L Cichero JA. Dysphagia management: does structured training improve the validity and reliability of cervical auscultation? Int J Speech Lang Pathol. (2021) 24:1–11. 10.1080/17549507.2021.1953592 34328050

[13] Peng JM Qian CY Yu XY Zhao MY Li SS Ma XC. Does training improve diagnostic accuracy and inter-rater agreement in applying the Berlin radiographic definition of acute respiratory distress syndrome? A multicenter prospective study. Crit Care. (2017) 21:12. 10.1186/s13054-017-1606-4 28107822 PMC5251343

[14] Bowton E Field JR Wang S Schildcrout JS Driest SLV Delaney JT. Biobanks and electronic medical records: enabling cost-effective research. science translational medicine. (2014) 6:234cm3–cm3. 10.1126/scitranslmed.3008604 24786321 PMC4226414

[15] Dean BB Lam J Natoli JL Butler Q Aguilar D Nordyke RJ. Review: use of electronic medical records for health outcomes research: a literature review. (2009) 66:611–38. 10.1177/1077558709332440 19279318

