# Supplementary material for: Development and evaluation of a survey instrument to assess veterinary medical record suitability for multi-center research studies
Source: Front Vet Sci. 2022 Aug 5;9:941036. doi: 10.3389/fvets.2022.941036 (PMC9389294; doi:10.3389/fvets.2022.941036)
Supplement: Supplementary file 1 [file Data_Sheet_1.docx]

Supplementary Materials

Supplemental Table 1: Survey instrument used in Study 1 with branching logic steps included.

| **Question** | **Question Category** | **Response type** | **Quality Score Points** |
| --- | --- | --- | --- |
| VEMR entry completed by (enter your initials) | Administrative | Text | N/A |
| Is the VEMR record at least 90% digital (typed, not handwritten) and in the appropriate format (pdf, txt, doc)? | Qualification | Y/N | N/A |
| Is the record legible (easy to read) and in English? | Qualification | Y/N | N/A |
| Dog’s Name | Verification | Text | N/A |
| Owner’s Last Name | Verification | Text | N/A |
| Owner’s Zip Code | Verification | Text | N/A |
| Owner’s Phone Number | Verification | Text | N/A |
| Does the VEMR match the participant record based on ALL of the following:   - Dog name (slight variations in spelling allowed)  - Owner name (slight variations in spelling allowed)  - Owner phone number or ZIP code - one or the other required | Verification | Y/N | N/A |
| *>>> Branching Logic -* If no to question 8 - What criteria DO MATCH?   - Dog name (slight variations in spelling allowed)  - Owner name (slight variations in spelling allowed)  - Owner contact information (address or phone number)  - None | Verification | Select all that apply | N/A |
| *>>> Branching Logic -* If no to question 8 - Manual verification completed by | Verification | Text | N/A |
| Dog’s date of birth or age (date of birth is preferred, so please enter DOB if it is available)  - Dog's date of birth  - Dog's age | Quality Score | Select One | 2 |
| *>>> Branching logic -* If Dog's date of birth selected, provide date of birth | Quality Score | Calendar entry | N/A |
| *>>> Branching logic -* If Dog's age selected   Dog’s age at data entry time - Years  Dog’s age at data entry time - Years | Quality Score | Drop-down number list | N/A |
| Dog’s sex  - Male  - Female  - Not Found | Quality Score | Select one | 2* |
| Is Dog Spayed/Neutered | Quality Score | Y/N |  |
| Is the dog a pure breed or mixed breed? | Administrative | Select one | N/A |
| *>>> Branching logic*  If pure breed - What breed is the dog?  If mixed breed - What is the dog's first breed? What is the dog's second breed? | Administrative | Drop-down | N/A |
| Date of spay/neuter | Quality Score | Calendar entry | 1~ |
| Dog’s weight in pounds (lb) or kilograms (kg) (or Not Found) | Quality Score | Select one | N/A |
| *>>> Branching logic -* Weight in pounds or Weight in kilograms | Quality Score | Text | 2 |
| Date of last vaccination (Do not enter based on reminders or due dates. Only enter if last vaccine is present on the record) | Quality Score | Calendar entry | 1~ |
| Results of any diagnostic test are present (heartworm test, fecal float, bloodwork, etc.). Select no if a test is indicated but no results are in the record. | Quality Score | Y/N | 1~ |
| Date of last sedated or anesthetized surgery or dental procedure | Quality Score | Calendar entry | 1~ |
| Any pharmacy purchases on the record? (Please be sure not to “double count” items that may have already been counted elsewhere on this form.) | Quality Score | Y/N | 1~ |
| Any preventative health care items on the record? | Quality Score | Y/N | 1~ |
| Which type of Body Condition Scoring was used (out of 5, out of 9, Unknown) | Administrative | Select one | N/A |
| *>>> Branching logic -* Provide Body Condition Score | Administrative | Text | N/A |
| Diagnoses at last visit (please separate with a comma). | Administrative | Text | N/A |
| Most recent entry is within the past 12 months? | Quality Score | Y/N | 1~ |
| Earliest entry is within 1 year of date of birth? | Quality Score | Y/N | 1~ |
| Total number of visits recorded (where dog was seen by a veterinarian) | Quality Score | Text | 1^ |
| Patient ID (if available) | Administrative | Text | N/A |
| Time to review & input record | Administrative | Text | N/A |

| * Both sex and spay/neuter status must be present to gain quality score points. |
| --- |
| ~ Point gained if answer is "Yes" or data is otherwise available (not missing or unknown). |
| ^Point gained if total number of visits recorded is >/= age * 0.5 |

Supplemental Table 2: Matrix arrangement of dyads that completed surveys in Study 1 and Study 2. Each of the 4 individuals completing the survey (A-D) were assigned as “reviewer 1” and “reviewer 2” to create 12 dyads.

|  | Reviewer 2 | | | | |
| --- | --- | --- | --- | --- | --- |
|  |  | A | B | C | D |
| Reviewer 1 | A | - | AB | AC | AD |
|  | B | BA | - | BC | BD |
|  | C | CA | CB | - | CD |
|  | D | DA | DB | CD | - |

Supplemental Table 3: Survey instrument used in Study 2 with branching logic steps included.

| **Question** | **Question Category** | **Response type** | **Quality Score Points** |
| --- | --- | --- | --- |
| VEMR entry completed by (enter your initials) | Administrative | Text | N/A |
| Is the current primary care clinic [populate clinic name] using an electronic record? (If the answer is NO, please complete this question and then enter your time to review and save this record as COMPLETE.)  >>> If NO - FAIL | Qualification | Y/N | N/A |
| Is the entire record in the appropriate file format? (If NONE of the files are in an appropriate format, please complete this question and then enter your time to review and save this record as COMPLETE.) | Qualification | Y/N | N/A |
| *>>> Branching logic*  If Yes - Please check which file type(s) were uploaded (check all that apply).  - pdf  - doc, txt, rtf  If No - Please check which file type(s) were uploaded (check all that apply).  - pdf  - jpg, png, tif  - zip  - doc, txt, rtf | Qualification | Select all that apply | N/A |
| Is the record legible (easy to read) and in English? (If the answer is NO, please complete this question and then enter your time to review and save this record as COMPLETE.)  >>> If NO - FAIL | Qualification | Y/N | N/A |
| Dog’s Name *Manually enter “Data N/A” if no data provided* | Verification | Text | N/A |
| Owner’s Last Name *Manually enter “Data N/A” if no data provided* | Verification | Text | N/A |
| Owner’s Zip Code *Manually enter “Data N/A” if no data provided* | Verification | Text | N/A |
| Owner’s Phone Number *Manually enter “Data N/A” if no data provided* | Verification | Text | N/A |
| Does the VEMR match the participant record based on ALL of the following:   - Dog name (slight variations in spelling allowed)  - Owner name (slight variations in spelling allowed)  - Owner phone number or ZIP code - one or the other required | Verification | Y/N | N/A |
| *>>> Branching Logic -* If no to question 9 - What criteria DO MATCH?   - Dog name (slight variations in spelling allowed)  - Owner name (slight variations in spelling allowed)  - Owner contact information (address or phone number)  - None | Verification | Select all the apply | N/A |
| *>>> Branching Logic -* If no to question 9 - (If the record is unverified, please either manually verify the record and continue processing, or enter your time to review and save this record as UNVERIFIED.) Manual verification completed by | Verification | Text | N/A |
| What is the date for the most recent electronic medical record entry available? | Administrative | Calendar entry | N/A |
| Has the dog been seen by a veterinarian at [fs_pcvet_cinic] within two years of the date the record was uploaded? (If the answer is NO, please complete this question and then enter your time to review and save this record as COMPLETE.)  >>> If NO - FAIL | Qualification | Y/N | N/A |
| Most recent entry is within the past 12 months? | Quality Score | Y/N | 1~ |
| Is the most recent electronic entry within the past 12 months? Entry can be of any entry type: vet visit, tech visit, pharmacy purchase, phone call, etc. | Qualification | Y/N | N/A |
| Patient ID (if available) *Manually enter “Data N/A” if no data provided* | Administrative | Text | N/A |
| Client ID (if available) *Manually enter “Data N/A” if no data provided* | Administrative | Text | N/A |
| Dog’s date of birth or age (date of birth is preferred, so please enter DOB if it is available)  - Dog's date of birth  - Dog's age | Quality Score | Select One | 2 |
| *>>> Branching logic -* If Dog's date of birth selected, provide date of birth | Quality Score | Calendar entry | N/A |
| *>>> Branching logic -* If Dog's age selected   Dog’s age at data entry time - Years  Dog’s age at data entry time - Years | Quality Score | Drop-down number list | N/A |
| Dog’s sex | Quality Score | Select one | 2* |
| Is Dog Spayed/Neutered (Yes, No, Unknow) | Quality Score | Y/N | N/A |
| Is the dog a pure breed or mixed breed? | Administrative | Select one | N/A |
| *>>> Branching logic*  If pure breed - What breed is the dog?  If mixed breed - What is the dog's first breed? What is the dog's second breed? | Administrative | Drop-down | N/A |
| Is the date of spay/neuter available? | Quality Score | Y/N |  |
| *>>> Branching logic -* If yes - provide date | Quality Score | Calendar entry | 1~ |
| Most recent weight in pounds (lb) or kilograms (kg) (or Not Found) | Quality Score | Select one | N/A |
| *>>> Branching logic -* Weight in pounds or Weight in kilograms | Quality Score | Text | 2 |
| Is the date of last vaccination present? (Do not enter based on reminders or due dates. Only enter if last vaccine is present in the record). | Quality Score | Y/N | N/A |
| *>>> Branching logic* - If YES - provide date | Quality Score | Calendar entry | 1~ |
| Is the date of last RABIES vaccination present? (Do not enter based on reminders or due dates. Only enter if last vaccine is present in the record). | Administrative | Y/N | N/A |
| *>>> Branching logic* - If YES - provide date | Administrative | Calendar entry | N/A |
| Results of any diagnostic test are present (heartworm test, fecal float, bloodwork, x-rays, urinalysis, ultrasound, etc.).  - All or some listed diagnostic results are present.  - No listed diagnostic results are present.  - No diagnostics appear to have been done. | Quality Score | Select one | 1~ |
| Is there a sedated or anesthetized surgery or dental procedure, not including, spay or castration, on record?  - Yes and the date is present  - Yes but no date is available  - No procedure on record | Quality Score | Select one | N/A |
| *>>> Branching logic -* If Yes - [Calendar entry] | Quality Score | Calendar entry | 1~ |
| Any pharmacy purchases on the record? (Written prescriptions for non-preventative items are included.) | Quality Score | Y/N | 1~ |
| Any preventative health care items on the record? (Written prescriptions for preventatives items are included.) | Quality Score | Y/N | 1~ |
| Which type of Body Condition Scoring was used   - Provide Body Condition Score out of 5  - Body Condition Score out of 9  - Score available but unit unknown  - Not Found | Administrative | Select one | N/A |
| *>>> Branching logic -* If available, provide Body Condition Score | Administrative | Text | N/A |
| Diagnoses at last visit (please separate with a comma). *Manually enter “Data N/A” if no data provided* | Administrative | Text | N/A |
| Total number of visits recorded (where dog was seen by a veterinarian) | Quality Score | Y/N | 1^ |
| What is the date of the earliest medical record entry of any kind (including handwritten) available? | Administrative | Calendar entry | N/A |
| What is the date of the earliest electronic medical record entry available? | Administrative | Calendar entry | N/A |
| Earliest entry is within 1 year of date of birth? | Quality Score | Y/N | 1~ |
| Additional records potentially available | Administrative | Y/N | N/A |
| Time to review & input record | Administrative | Text | N/A |

| * Both sex and spay/neuter status must be present to gain quality score points. |
| --- |
| ~ Point gained if answer is "Yes" or data is otherwise available (not missing or unknown). |
| ^Point gained if total number of visits recorded is >/= age * 0.5 |
